# Supplementary material for: Focused ultrasound-induced cell apoptosis for the treatment of tumours
Source: PeerJ. 2024 Aug 21;12:e17886. doi: 10.7717/peerj.17886 (PMC11344538; doi:10.7717/peerj.17886)
Supplement: Supplemental Information 1 — A list of all used keywords [file peerj-12-17886-s001.docx]

**Appendix**

**Keywords in document retrieval**

| cancer treatment | ultrasound |
| --- | --- |
| thermal effects | mechanical effect |
| Apoptosis | Death receptor pathway |
| Mitochondrial apoptosis pathway | non-cumulative effect |
| apoptosis regulators | high-intensity focused ultrasound(HIFU) |
| necrosis | CD95 |
| TNF | NF-κB |
| mitochondrial membrane potential(MMP) | mitochondrial outer membrane (MOMP) |
| Endoplasmic reticulum stress（ERS） | CHOP |
| phosphatidylserine (PtdSer) | ROS |
| Focused ultrasound（FUS） | UTMD |
| Microbubble | Therapical gas |
| Endoplasmic reticulum stress signaling pathway | radiotherapy |
| programmed cell death | chemotherapy |
| low-intensity focused ultrasound(LIFU) | clearance of apoptotic cells |
| TRAIL | Macrophage |
| Bcl-2 family proteins | unfolded protein response (UPR) |
